# Supplementary material for: Effects of Induced Exosomes from Endometrial Cancer Cells on Tumor Activity in the Presence of Aurea helianthus Extract
Source: Molecules. 2021 Apr 12;26(8):2207. doi: 10.3390/molecules26082207 (PMC8068874; doi:10.3390/molecules26082207)
Supplement: Supplementary file 1 [file molecules-26-02207-s001.zip › S2.pdf]

Gene		Primer Sequence<br>Forward (F) 5′->3′, Reverse (R) 5′->3′
SCC<br>(squamous cell carcinoma antigen)	F	ACACTGGTTCTTGTGAACGC
	R	GCCTGTACATCCTTGTTTGGC
UGF<br>(urinary gonadotropin fragment)	F	ATGTGCGCTTCGAGTCCATC
	R	AGAAAGACCCGCCAGAGTGC
Il-2 receptor<br>(interleukin 2 receptor)	F	CAGTTCGCCGCATCCTTCT
	R	TAAGTATTGGGCTGGCGTGT
mTORC2<br>(mechanistic target of rapamycin complex 2)	F	AGTACGAGGGCGGAATGACA
	R	GCCACCACCTCTGGATTCTG
NF-κB (P50)<br>(nuclear factor kappa-light-chain-enhancer of activated B cells)	F	CGGAGCCCTCTTTCACAGTT
	R	TTCAGCTTAGGAGCGAAGGC
NF-κB (P52)	F	AGGTGCTGTAGCGGGATTTC
	R	AGCGGCACTGTATAGGGCAGA
ABCB1<br>(ATP Binding Cassette Subfamily B Member 1)	F	TCTTGTCCAAACTGCCTGTGA
	R	TAATTGTGCCTCACCCCACC
PTGS2<br>(prostaglandin-endoperoxide synthase 2)	F	TGCGAATGTTTCAGTGCCTC
	R	CCATTTCATGAAGGGCCAGT
ULK1<br>(unc-51 like autophagy activating kinase 1)	F	GCGGCTCTTTTGTTTCTCCG
	R	CTTGCGGGAGAACTCGAACT
TRIM28<br>(tripartite motif containing 28)	F	GGAGCTGAGAGGGGAATCAC
	R	AGAAGCACTGTTGCTTGCAC
ATP5A1	F	GGTCGTGTAGTTGATGGCCCT
	R	GAGGTCAGAACCGAACTGGG
HSP17B12<br>(hydroxysteroid 17-beta dehydrogenase 12)	F	CTCCTGGATTCATTCACTCGCT
	R	AGTACGAAATACGCAGGGCT
ESR2<br>*(*Estrogen receptor 2)	F	CCATGAGGGGCACCAATGTA
	R	CAAATGAACAGGCAAAGCCCC
STRN3 (striatin 3)	F	GCACAGAATGGGCTGAACCA
	R	CCGATGTGGGCCCTAAATGT
AR (Androgen receptor)	F	AGCCTGTAAGCAAACGATGGA
	R	GAGCTGGGGTGGGGAAATAG
GAPDH	F	AGTCTGTTATAACCCAGACGAG
	R	GCATCACAACCAATAGGTGTGA
