# Supplementary figures and images for: Effects of Induced Exosomes from Endometrial Cancer Cells on Tumor Activity in the Presence of Aurea helianthus Extract
Source: Molecules. 2021 Apr 12;26(8):2207. doi: 10.3390/molecules26082207 (PMC8068874; doi:10.3390/molecules26082207)

Figure 1.

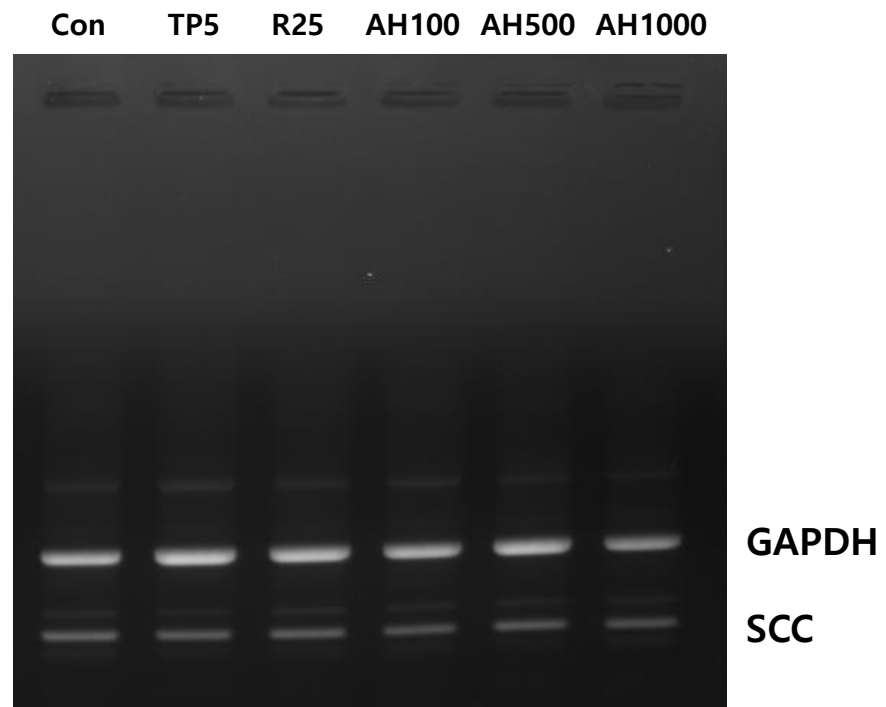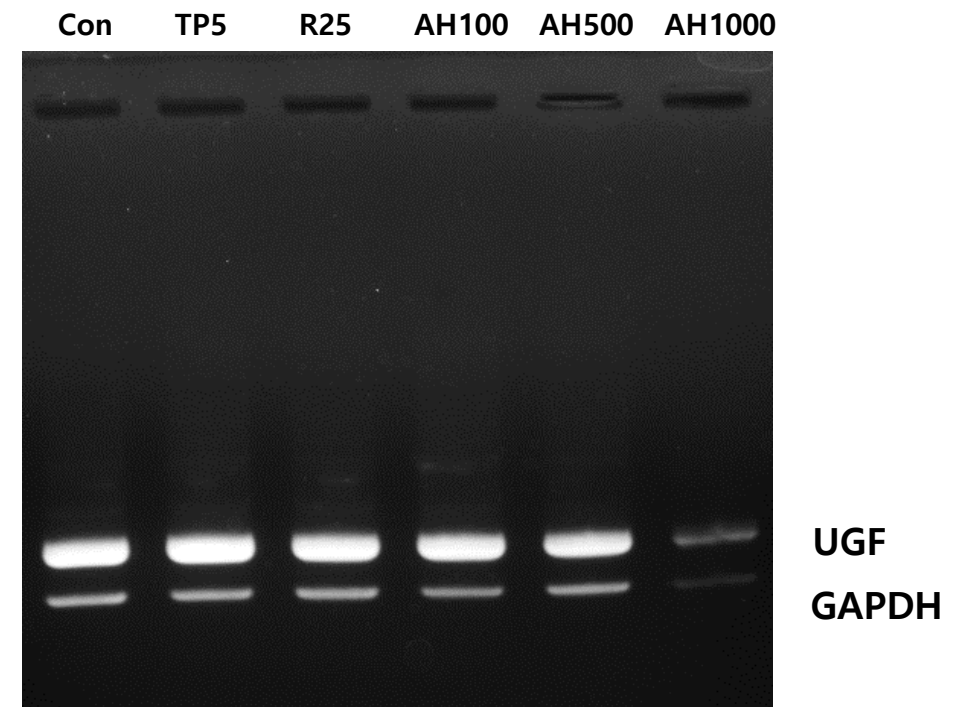

Figure 3.

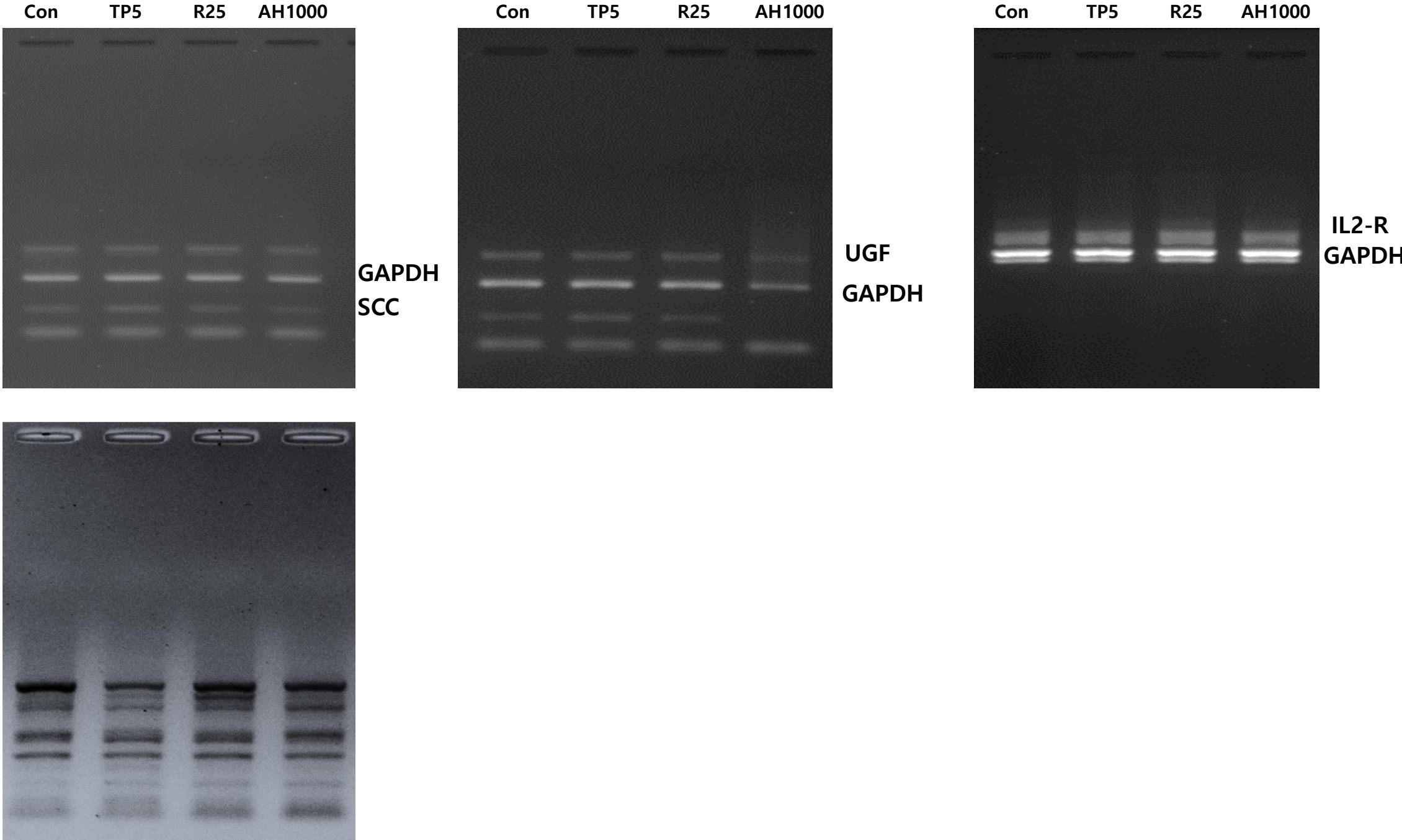

Fig. 4

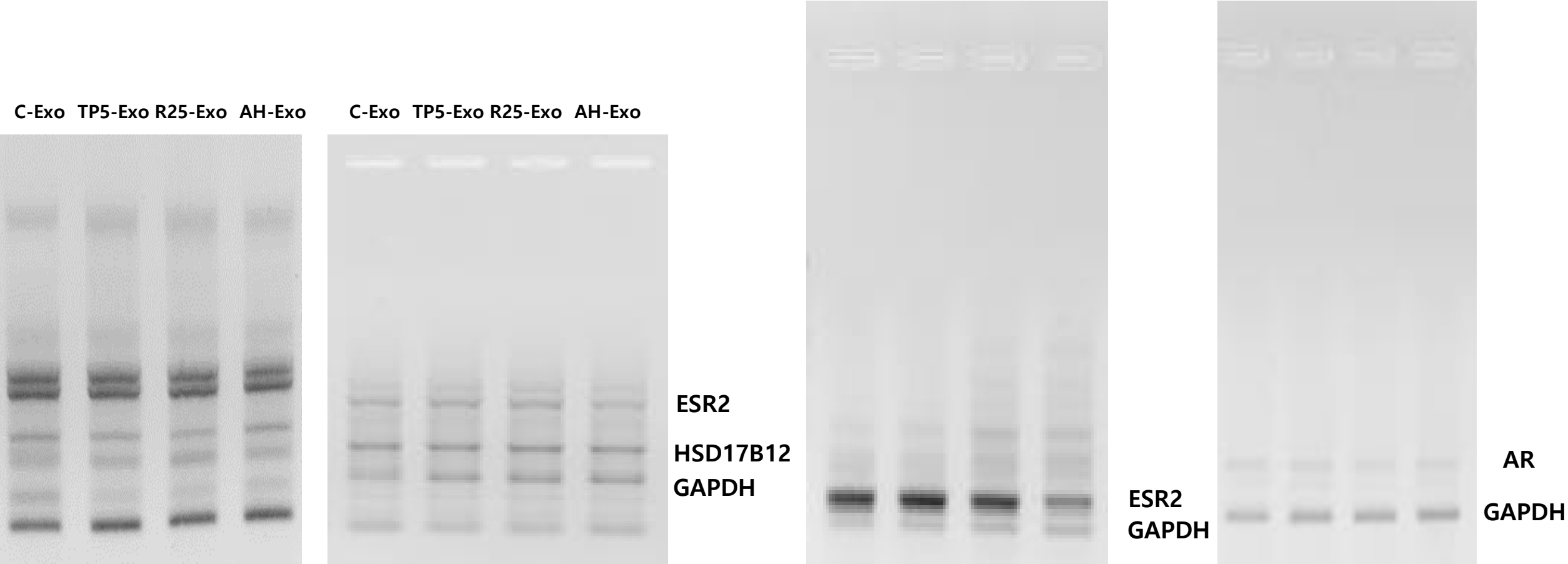

Supplement: Supplementary file 1 [file molecules-26-02207-s001.zip › S1.pdf]
